# Molecular detection of rifampicin-resistant *Mycobacterium tuberculosis* by polymerase chain reaction in Ethiopia: a systematic review and meta-analysis

**DOI:** 10.3389/fmed.2024.1319845

**Published:** 2024-06-07

**Authors:** Molla Yigzaw Birhanu, Getamesay Molla Bekele, Selamawit Shita Jemberie

**Affiliations:** ^1^Department of Public Health, College of Health Sciences, Debre Markos University, Debre Markos, Ethiopia; ^2^Department of Gynecology and Obstetrics, School of Medicine, Debre Markos University, Debre Markos, Ethiopia; ^3^Department of Midwifery, College of Health Sciences, Debre Markos University, Debre Markos, Ethiopia

**Keywords:** rifampicin-resistant, prevalence, associated factors, tuberculosis, Ethiopia

## Abstract

**Introduction:**

Tuberculosis is a contagious bacterial disease caused by *Mycobacterium tuberculosis*. The emergence and spread of drug-resistant strains of *M. tuberculosis* in both developing and developed countries has made diagnosis, treatment, and control of tuberculosis more difficult. The PCR assay, which is a fast and sensitive technique and an alternative method for detecting multidrug-resistant tuberculosis, is used to determine rifampicin (RIF) resistance. There is no single figure in Ethiopia that represents rifampicin-resistant tuberculosis and that is why this study was conducted to overcome the inconsistency of the results of the previous studies.

**Methods:**

Studies were researched from five major electronic databases. Studies which were cross-sectional in design, published, and written in English were included. The data were extracted using Microsoft Excel, and the data were managed and analyzed using Stata™ Version 17.0 statistical software. The Forest plot was used to check the presence of heterogeneity. The publication bias, meta-regression, and subgroup analysis were used to find out the source of heterogeneity. A random effect analysis model was used to pool the prevalence of RR TB from primary studies, and associated factors of RR among TB patients were identified using Meta regression. The presence of association was reported using OR with 95% CI.

**Results:**

The overall pooled prevalence of tuberculosis was 14.9% (95% CI: 13.34, 16.46), of these approximately 7.48% (95% CI: 6.30, 8.66) showed rifampicin-resistant tuberculosis in Ethiopia. Among the computed variables, 2.05% living with HIV1.39 (95%CI: 1.13, 1.72) and having a history of TB treatment (95%CI: 1.34, 3.15) were identified as significant factors associated with RR TB in Ethiopia.

**Conclusion:**

Drug-resistant TB is one of the prevalent emerging infectious diseases among TB patients, which affects approximately one out of every thirteen TB patients. Having TB-HIV coinfection and a history of prior TB treatment were identified as significant factors associated with RR TB. To prevent and control RR TB, patients should complete their follow-up course; the health professionals should educate the actions taken by the patients when they experience drug toxicity and side effects; and the Minister of Health should initiate telemedicine and recruit tracers to overcome TB patients’ default and have good drug adherence and retention after initiation of the treatment.

## Introduction

Tuberculosis is a communicable bacterial disease caused by *Mycobacterium tuberculosis* (1). The bacteria primarily affect the lungs to cause pulmonary tuberculosis; however, the bacteria can affect other organs. It is one of the top ten causes of death worldwide, ranking even higher than the human immunodeficiency virus (2). Nowadays, diagnosis, treatment, and control of tuberculosis are made more difficult by the emergence and increase in the spread of drug-resistant strains of *M. tuberculosis* found in both developing and developed countries (3–5).

Multidrug-resistant tuberculosis (MDR-TB) is defined as the presence of a strain of *Mycobacterium tuberculosis* that is resistant to at least rifampicin and/or isoniazid (6). Rifampicin (RIF) is the most potent first-line anti-tuberculosis antibiotic, which is essential to the success of the current tuberculosis chemotherapy regimen. Rifampicin-resistant (RIF) necessitates treatment extension and may lead to multidrug-resistant tuberculosis in the future (7, 8). RIF is caused by the substitution of a small number of highly conserved amino acids encoded by rpoB, the gene for the β subunit of *Mycobacterium tuberculosis* RNA polymerase (9). RIF caused fewer and more expensive therapeutic options, prolonged treatment, increased toxicity, and poorer clinical outcomes (10, 11). Determining RIF-resistant strains using the gene X-pert assay, a rapid and sensitive technique, is an alternative approach to detecting multidrug-resistant tuberculosis (MDR-TB) (12, 13). This is because more than 90% of RIF-resistant strains are also resistant to isoniazid (INH) (7–10), and this could be explained by the rate of spontaneous mutation rate, with INH target gene having a 100-fold higher rate of mutation than RIF target gene (14).

In Ethiopia, the burden of tuberculosis is high, and the diagnostics are woefully inadequate, with most cases being diagnosed using conventional methods. The emergence of rifampicin-resistant, combined with poor environment and living conditions, poses a significant threat to public health and safety in the country (15).

Since 2014, the PCR assay has been used for the tuberculosis diagnostic technique in referral hospitals and regional laboratories in Ethiopia, and it is now being used in health facilities in accordance with the WHO recommendations. Sputum microscopy was the most commonly used laboratory diagnostic technique for tuberculosis. Furthermore, the magnitude of rifampicin-resistant tuberculosis has not been addressed extensively in Ethiopia using the newly developed diagnostic method PCR. As a result, the goal of this study was to provide a pooled prevalence of tuberculosis and rifampicin resistance among patients tested in Ethiopia using the PCR molecular method.

*Research question*: What is the prevalence and associated factors of rifampicin-resistant tuberculosis among Ethiopian tuberculosis patients?

*Condition*: Rifampicin-Resistant Tuberculosis.

*Context*: Ethiopia.

*Population*: All Ethiopian presumptive tuberculosis patients.

## Methods

### Data source and searching strategy

The Preferred Reporting Items for Systematic Review and Meta-Analysis (PRISMA) guideline was used to report this review (16) (Supplementary File1). We systematically searched five major databases (PubMed/MEDLINE, CINAHL, EMBASE, Google Scholar, and Science Direct) for studies similar with our topic of interest. Moreover, related studies were explored by searching the reference lists of eligible studies or the reference of reference. The different search strategies were applied depending on the database requirements. The two writers (MYB and SSJ) conducted the searching operations independently. Endnote X9 was used to retrieve and manage studies that were found through a systematic search. In the search, strategy different keywords/text words and medical subject headings were applied accordingly. This is an example for search strategy applied in PubMed database: “(((((((Rifampicin [Text Word]) OR (prevalence [Text Word])) OR (associated factors [Text Word])) OR (Rifampicin resistant [Text Word])) OR (tuberculosis [MeSH Terms])) OR (Rifampicin [MeSH Terms])) AND (Ethiopia [Text Word])) AND (Ethiopia [MeSH Terms]).” The searching strategy was performed from 28 July 2023 to 20 August 2023.

### Study selection criteria

#### Inclusion criteria

All studies conducted on rifampicin-resistant tuberculosis in Ethiopia were included.

### Exclusion criteria

Those studies whose full-length were not found after two email exchanges with the corresponding authors were excluded.

*Design*: A systematic review and meta-analysis.

*Publication status*: All published.

*Language*: English language.

### Screening procedure

Two authors (MYB and GMB) independently screened all titles/abstracts found in electronic databases. Disagreements were resolved through dialog. Two authors (MYB and GMB) independently screened all potentially significant full texts. Disagreements were resolved through dialog. A third author (SSJ) was consulted in the event of a disagreement.

### Data extraction process and quality assessment

A Microsoft Excel spreadsheet was used to create the data extraction form and extraction too. To ensure consistency, three authors (MYB, SSJ, and GMB) extracted data independently using a predefined eligibility criterion. The disagreements between authors were resolved through discussion. The study area, region, year of publication, sample size, prevalence of tuberculosis, rifampicin, and first author name were the data extracted from all included primary articles during extraction.

The Newcastle–Ottawa Quality Assessment Scale (NOQAS) (17) was used to evaluate the quality of the included primary studies based on study representativeness, adequate sample size, acceptable non-response rate, use of validated measurement tool, comparability of the study, description of outcome assessment, and use of appropriate statistical tests as parameters. In the quality assessment of the primary studies, those studies received seven out of ten were declared as high-quality studies (18, 19) (Additional file 2).

### Outcome variable and measures

In this systematic review and meta-analysis, the prevalence of rifampicin-resistant tuberculosis was the main outcome of interest. We included all studies examining the existence of rifampicin -resistant tuberculosis, and the presence or absence of diagnosis of tuberculosis was performed using PCR molecular technique among suspected patients. Using a PCR machine, the incubated sample was tested for *Mycobacterium tuberculosis* in the primary studies. Therefore, the prevalence of rifampicin-resistant tuberculosis was determined by pooling the effect size of the primary studies. The second outcome variable for this systematic review and meta-analysis was factors associated with rifampicin-resistant tuberculosis, which was measured using the study participants’ HIV status, history of tuberculosis treatment, and sex through Meta-regression analysis.

### Data management and analysis

The extracted data were exported to Stata™ version 17.0 software for further analysis. The metaprop stata command was used to compute the pooled estimate. Using a binomial distribution assumption, the standard errors were calculated from the reported estimates and population denominators. The presence or absence of heterogeneity between studies was checked using Cochran’s Q test and quantified using I-square statistics, and significant heterogeneity was found. Thus, a random-effect model based on DerSimonian and Laird was used to estimate the effect size (20). Accordingly, heterogeneity was classified as low, moderate, or high when the values of I-square were 25, 50, and 75%, respectively (19). Additionally, the dispersion of individual results in the forest plot was also used to evaluate the presence of heterogeneity visually. Egger’s linear regression test at a *p*-value of <0.05 was used to assess the presence of publication bias (20). To identify the possible sources of heterogeneity, additional statistical analyses such as subgroup analyses, publication bias, and meta-regression were performed. We conducted a subgroup meta-analysis using study regions, sample size (above mean vs. below the mean (5418)), and study period (before 2018 vs. after 2018). Finally, the results were presented in tables and Forest plots. The presence and absence of a significant association between rifampicin-resistant and independent variables were declared using a 95% confidence interval and odds ratio.

### Patient and public involvement statement

Patients were not directly involved in this study. Because this systematic review and meta-analysis were conducted using previous primary studies, no study participants were recruited, and participants were not involved in the recruitment and dissemination of the findings.

## Results

### Search results

**Figure 1:**
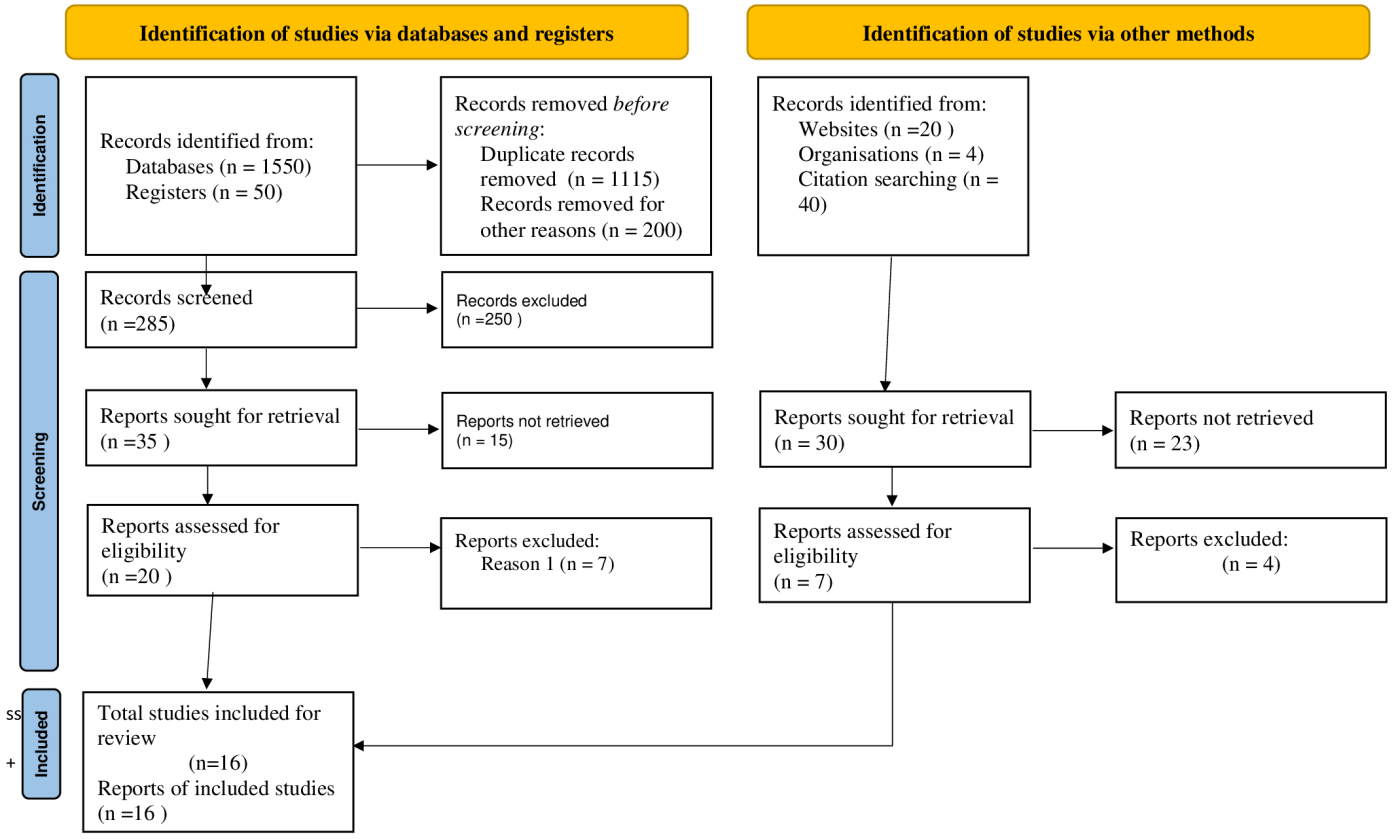
PRISMA flow diagram of the included studies for tinea capitis among schoolchildren in Ethiopia.

A total of 1,664 studies were discovered through electronic databases (PubMed, EMBASE, Google Scholar, and Science Direct) and other searches (such as organizational records and websites). Approximately 1,115 studies were excluded due to duplication, 351 studies were excluded due to differences in study setting/context (21–29), 105 studies were excluded due to differences in outcome interest (30–33), and 77 studies were excluded due to differences in the study population (27–29). Finally, 16 cross-sectional studies were identified as potentially relevant for inclusion in the current meta-analysis and systematic review (Figure 1).

### Characteristics of included studies

According to Table 1, approximately 16 studies with 99,429 presumptive tuberculosis patients met the inclusion criteria. This systematic review and meta-analysis included six regions and one city administration. Among them were the Amhara region (*n* = 8) (34–36, 39, 42, 45–47), the Oromia region (*n* = 1) (49), the SNNPR (*n* = 1) (48), Benishangul-Gumuz (*n* = 1) (44), Afar (*n* = 1) (43), Tigray (*n* = 1) (40), and Addis Ababa (*n* = 3) (37, 38, 41). The Amhara regional state had the study with the smallest and largest sample sizes of 170 and 26,656, respectively (Table 1).

**Table 1:** Characteristics of the studies included in this systematic review and meta-analysis.

Sn	Authors	Publication year	Region	Study area	Sample size	Prevalence
1	Araya Gebreyesus Wasihun et al. (34)	2021	Amhara	Dessie	26, 656	8.3
2	Sebsib Selfegna et al. (35)	2022	Amhara	Shewa	170	11.2
3	Wondemagegn Mulu et al. (36)	2017	Amhara	Debre Markos	505	10.3
4	Waganeh Sinshaw et al. (37)	2019	Addis Ababa	Addis Ababa	418	6.5
5	Balew Arega et al. (38)	2019	Addis Ababa	Addis Ababa	12,414	9.9
6	Feleke Mekonnen et al. (39)	2015	Amhara	Metema	248	5.7
7	Tsehaye Asmelash Dejene et al. (40)	2017	Tigray	Adwa	17,329	8.7
8	Shambel Araya et al. (41)	2022	Addiss Ababa	Merkato	12,685	9.8
9	Daniel Gebretsadik et al. (42)	2022	Amhara	Ataye	423	5.3
10	Gebremedhn Bizayen Gebrehiwet et al. (43)	2018	Afar	Dubti	384	4.3
11	Wakuman Taye et al. (44)	2021	Benishangul-Gumuz	Bale zone	301	4.35
12	Awoke Derbie et al. (45)	2016	Amhara	Debre Tabor	1922	9.3
13	Olifan Zewdie et al. (33)	2020	Oromia	Nekemte	2,300	5.5
14	Kefyalew N Jaleta et al. (46)	2017	Amhara	Gondar	1820	12
15	Tesfaye Andualem Demissie (47)	2021	Amhara	Motta	4,109	4.3
16	Kuma Diriba et al. (48)	2021	SNNP	Gedeo	17,745	5.1

### The pooled prevalence of rifampicin-resistant tuberculosis

**Figure 2:**
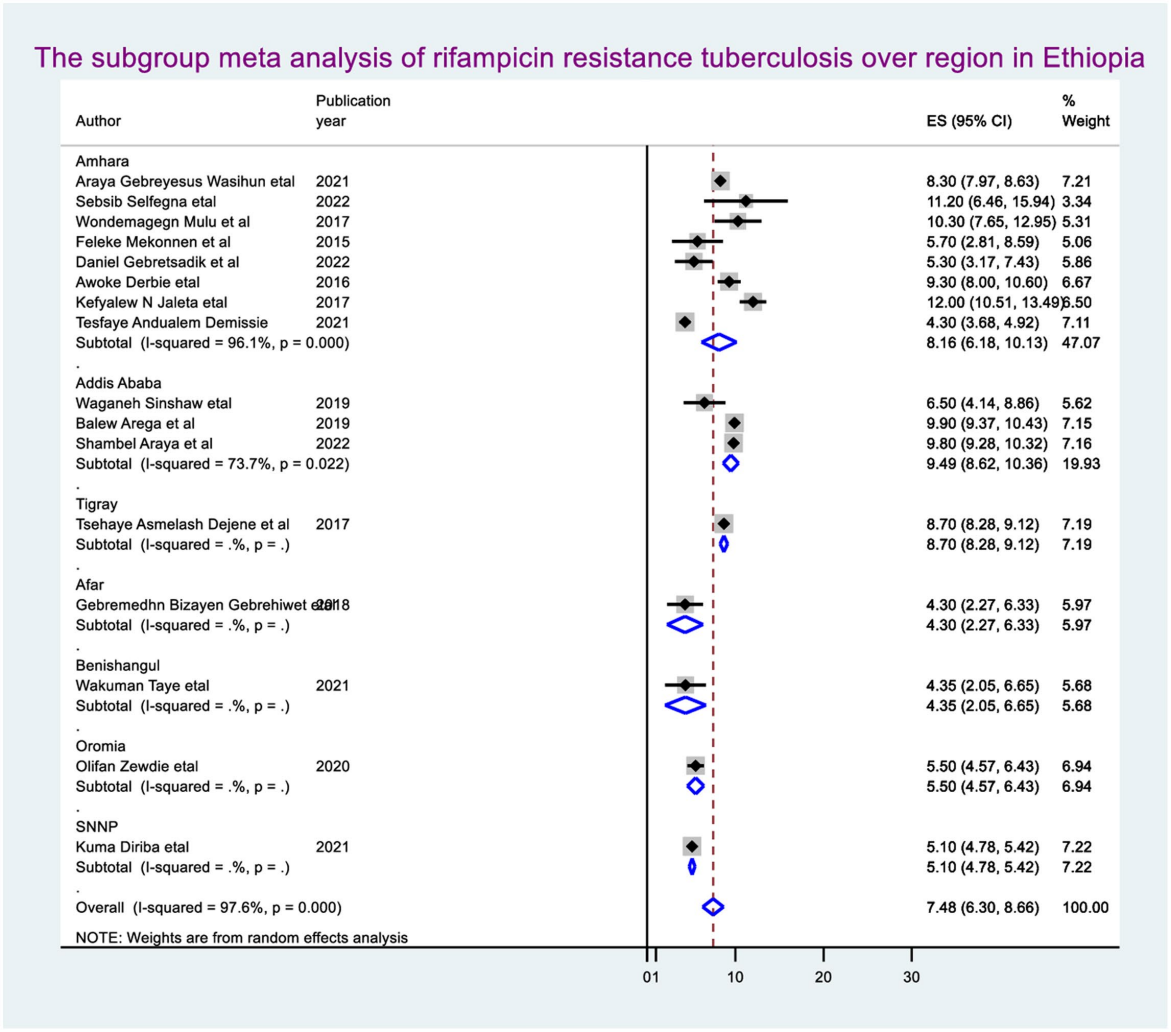
The pooled prevalence of rifampicin in Ethiopia.

By molecular detection, the overall prevalence of *Mycobacterium tuberculosis* was 14.9% (95% CI: 13.34, 16.46), approximately 7.48% (95% CI: 6.30, 8.66) showed rifampicin resistance in Ethiopia. When we looked at it by region, Addis Ababa city administration had the highest prevalence of rifampicin-resistant *Mycobacterium tuberculosis* (9.49, 95% CI: 8.62, 10.36), followed by Amhara regional state (8.16, 95% CI: 6.18, 10.13) (Figure 2).

### Subgroup meta-analysis

**Figure 3:**
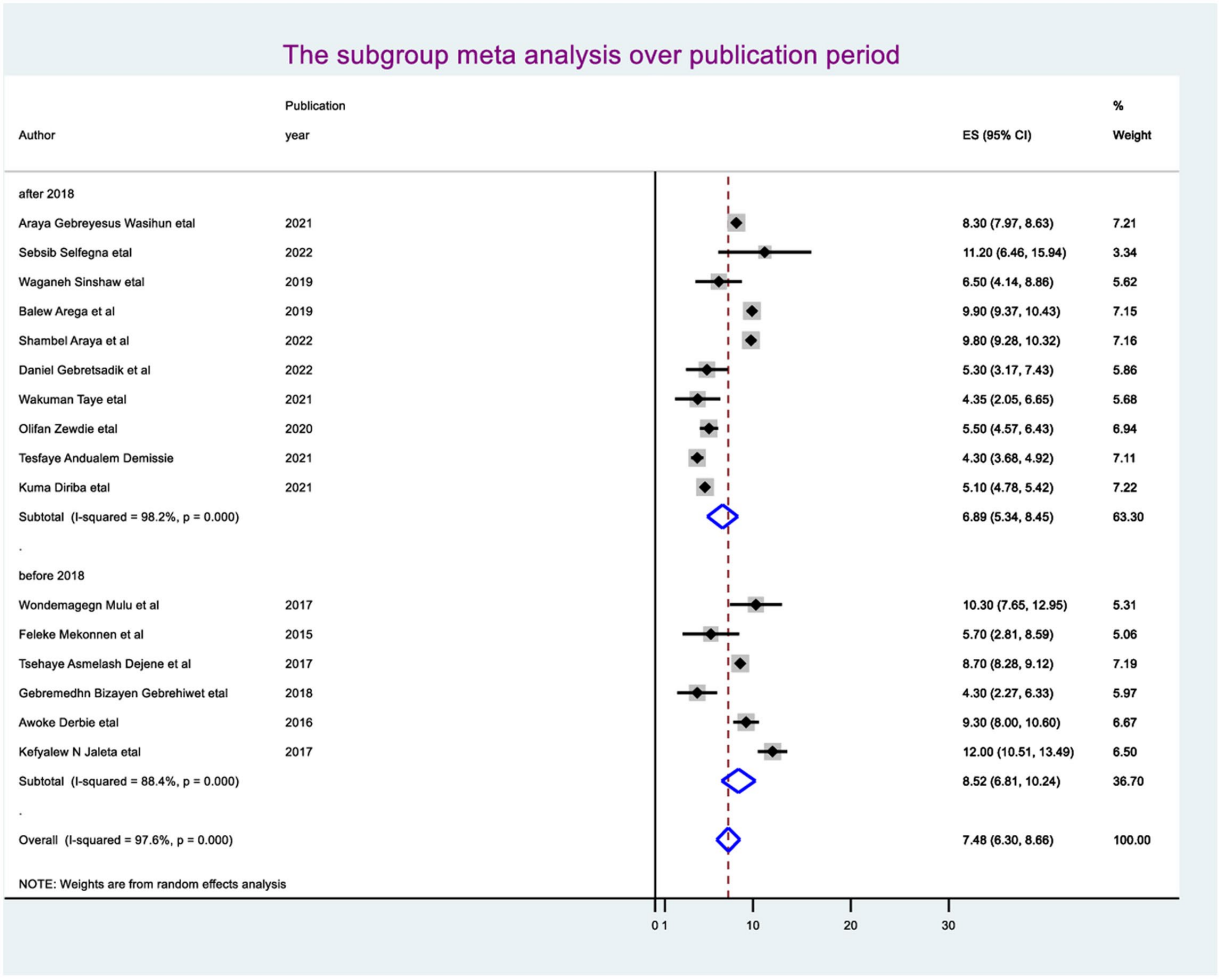
Subgroup analysis using sample.

**Figure 4:**
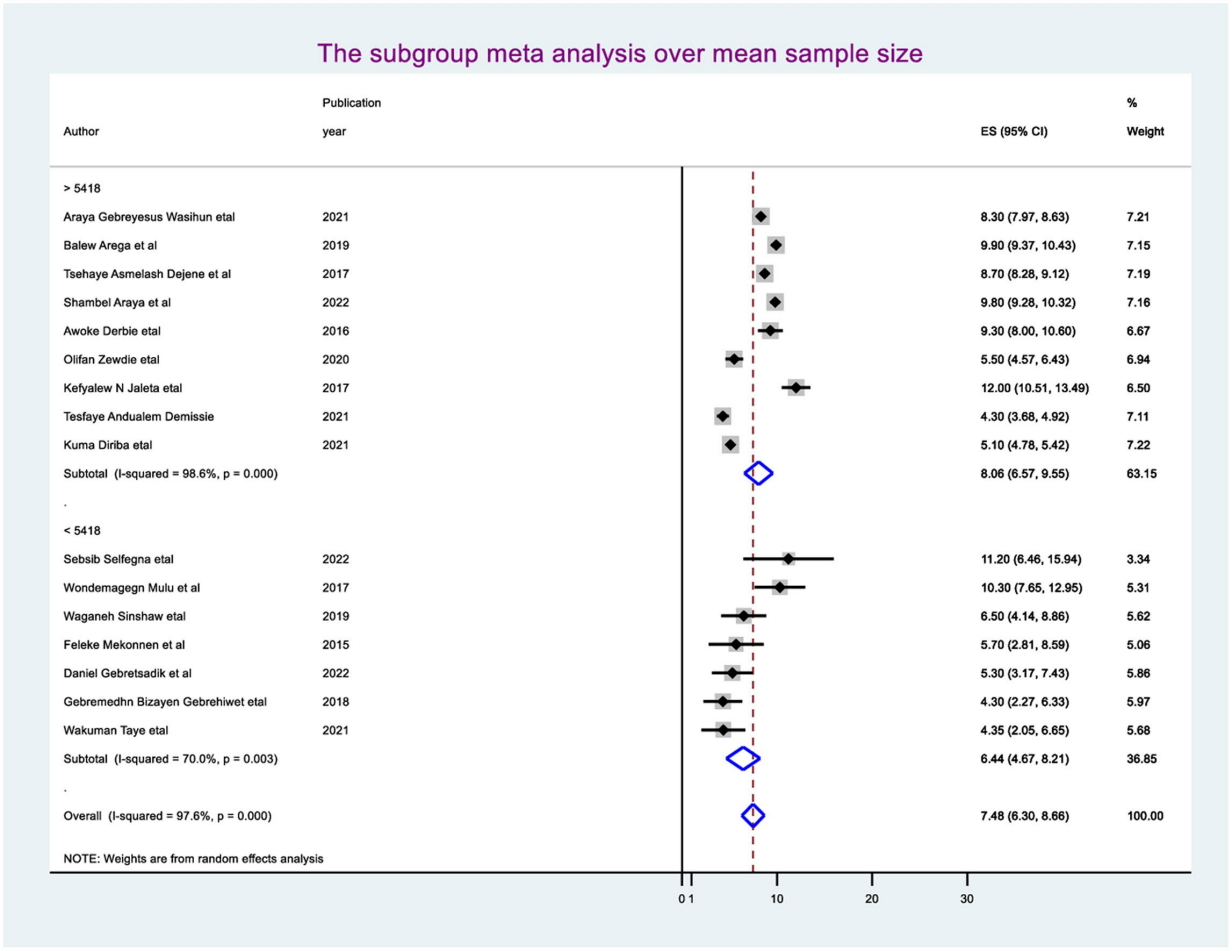
Subgroup analysis using the publication year.

The random-effect meta-regression model was used to identify the source of heterogeneity by conducting subgroup meta-analysis while taking into account study region, sample size, and publication year. As a result, there are no sources of heterogeneity identified using subgroup meta-analysis. However, there is strong evidence supporting the existence of heterogeneity, publication year before 2018 I^2^ = 88.4 with a *p*-value of 0.0001 and after I^2^ = 98.2 with a *p*-value of <0.0001 (Figure 3), and the sample size greater than the mean (>5,418) I^2^ = 98.6 and less than the mean sample size (< 5,418) I^2^ = 70.0 with a *p*-value of <0.0001 (Figure 4).

### Meta-regression

In random-effects meta-regression, the year of publication and sample size were used as covariates. The study found that sample size (*p* = 0.34) and publication year (*p* = 0.22) did not affect heterogeneity (heterogeneity was not explained by sample size or publication year) (Table 2).

**Table 2:** Meta-regression using year of publication and sample size for rifampicin-resistant *Mycobacterium tuberculosis* in Ethiopia.

logrr	Coefficient	Std. err.	*t*	P > |t|	[95% conf. interval]
Year of publication	0.00	0.01	0.99	0.34	−0.01,0 0.01
Sample size	−0.40	0.31	−1.29	0.22	−1.06,0.27
Constant	815.07	624.02	1.31	0.21	−533.16, 259.34

### Publication bias (bias detection)

**Figure 5:**
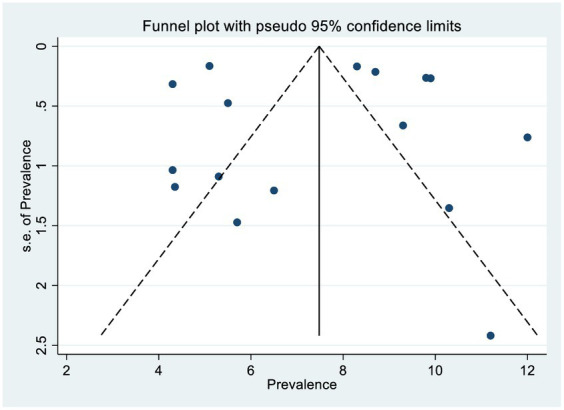
The funnel plot of rifampicin resistance in Ethiopia.

The scatter plots were symmetrical, and the funnel is wide at the bottom and narrow at the top, which indicates that the small study had no effects (free of publication bias) on the heterogeneity of rifampicin-resistant *Mycobacterium tuberculosis* (Figure 5), and then, we used the Egger linear regression test to objectively confirm the existence of publication bias. As a result, no statistically significant publication bias (*p* = 0.0001) was observed (Table 3).

**Table 3:** The table to check the publication bias objectively.

Std_Eff	Coefficient	Std. err.	*t*	P > |t|	[95% conf. interval]
Slope	7.39	0.88	8.36	0.000	5.499.28
Bias	0.37	2.66	0.14	0.89	−5.336.06

### Factors associated with rifampicin-resistant tuberculosis

Factors associated with rifampicin-resistant tuberculosis were determined using sex (including 10 studies) (38–41, 46, 47), history of tuberculosis treatment (including 10 studies) (38–41, 46, 47), and current HIV serostatus (including 10 studies) (38–41, 46, 47). Finally, rifampicin-resistant tuberculosis was significantly associated with having a history of tuberculosis treatment and living with HIV.

Being male had a 1.04 (95% CI: 0.87, 1.24), and living with HIV (being positive for HIV) had a 1.39 (95%CI: 1.13, 1.72).

**Figure 6:**
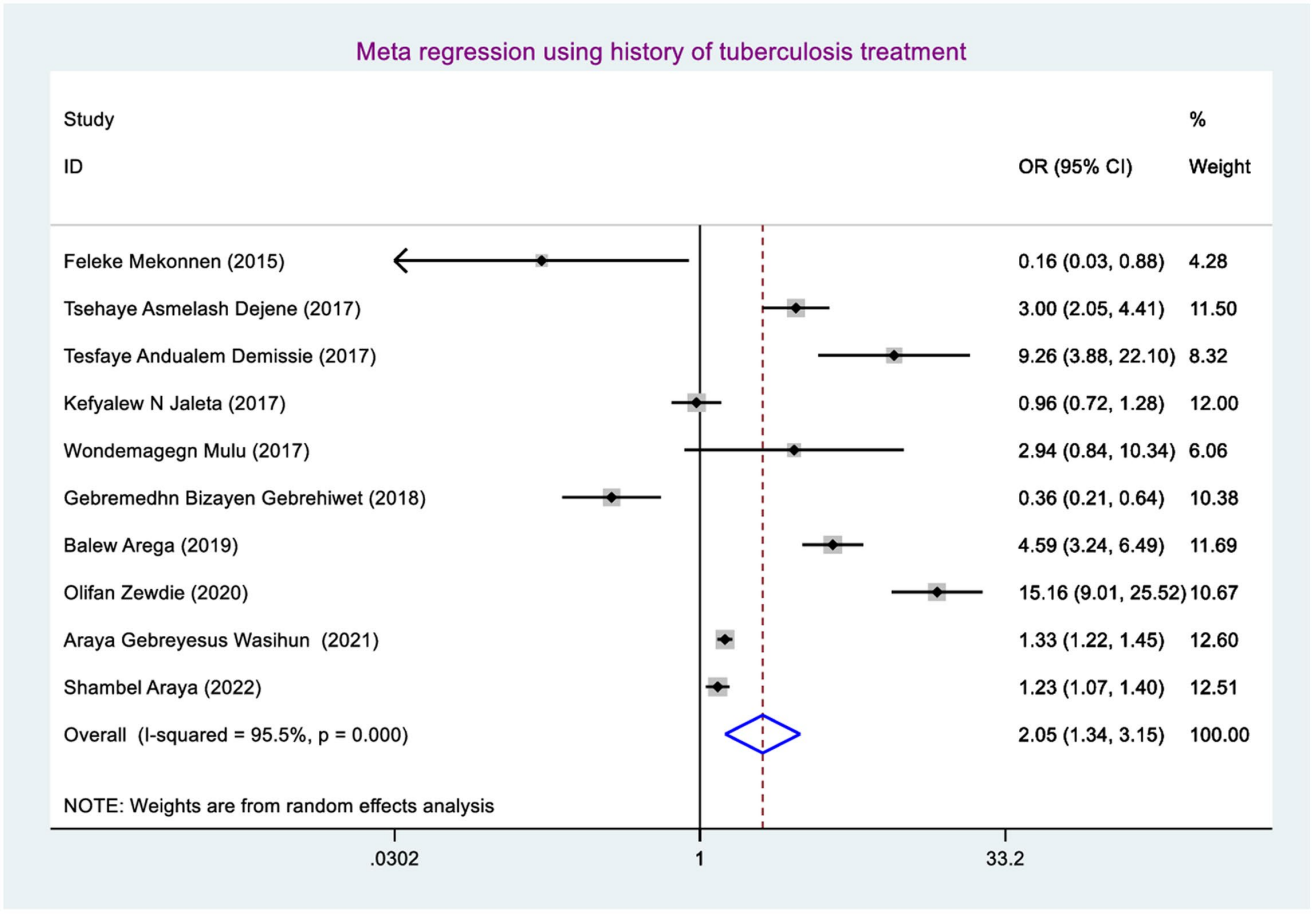
Meta-regression for a history of tuberculosis treatment.

The odds of developing rifampicin-resistant tuberculosis were 1.39 (95% CI: 1.13, 1.72) times higher among TB patients who have HIV-positive coinfection compared with their counterparts (Figure 6).

**Figure 7:**
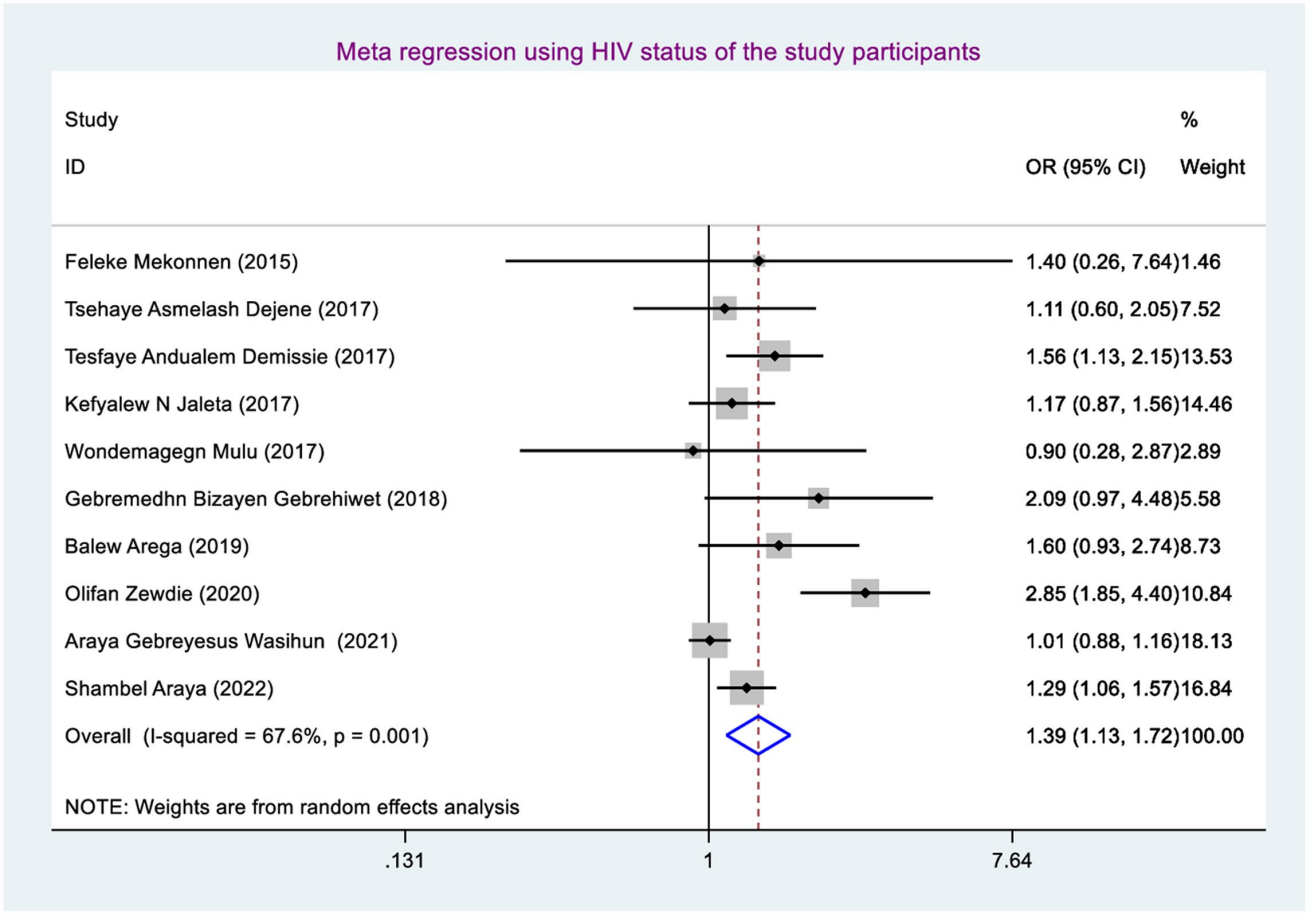
Meta-regression for coinfection of HIV in Ethiopia.

The odds of developing rifampicin-resistant tuberculosis among presumptive tuberculosis patients who had a history of tuberculosis treatment were 2.05 (95% CI: 1.34, 3.15) times higher when compared with those study participants who had no previous tuberculosis treatment (Figure 7).

## Discussion

This systematic review and meta-analysis incorporated 16 studies conducted between 2015 and 2022 with 99,429 tuberculosis patients. The sample size of each study ranged from 170 to 26,656 study participants. The study participants were selected using either a simple or systematic random sampling approach in each study.

By molecular detection, the overall pooled prevalence of TB was 14.9% (95% CI: 13.34, 16.46), and of these, 7.48% (95% CI: 6.30, 8.66) was the pooled prevalence of RR TB in Ethiopia. It means half of the TB patients in Ethiopia developed RR which is high to achieving end-TB- strategy by 2030 because of how we can achieve zero morbidity and mortality in the presence of a 3.0% secondary attack rate (50) and 39% mortality rate (51) with 7.48% existence of RR TB is keeping on. This could be due to the fact that TB patients delayed anti-TB drug initiation as well as no more close follow-up for supervising daily pill intake allowing poor adherence to their medication and resulting the comorbidities, mutated bacterial multiplication, immune deterioration, disease progression, and finally causing RR TB.

The prevalence of this study was higher than the prevalence of a study conducted in Botswana and Nigeria, which were 5.4 and 6%, respectively (52, 53). This could be because, in the current study, more study participants had TB-HIV coinfection, previous history of TB treatment (especially default), and less media exposure than what is happening in Nigeria and Botswana. As this study, finding supports, previous history of TB treatment and having TB-HIV coinfection were the significantly associated factors for developing RR TB. Therefore, the greater pooled prevalence of RR TB in Ethiopia might be a result.

In this study, the prevalence of RR tuberculosis was higher than the study findings in Botswana and Nigeria, which were 5.4 and 6%, respectively (52, 53). This could be because the current study had more study participants than the previous one, and as we know, a difference in the number of susceptible cases can affect the prevalence, which is why the prevalence of rifampicin resistance is higher in Ethiopia than in Botswana and Nigeria.

The odds of developing RRTB were 1.39 (95% CI: 1.13, 1.72) times higher among study participants who had TB-HIV coinfection than those who had not TB-HIV coinfection. This finding was in line with the study conducted in Nigeria (54). This might be due to the fact that study participants having TB-HIV coinfection may have overlapping of drug (toxicities, side effects), burden of pills, and drug–drug interaction, as well as HIV weakens the immune system, letting poor RIF adherence and resulting high mutated bacilli multiplication and disease progression and finally causing RR TB (55). That is why those study participants having TB-HIV coinfection had more chance of developing RR TB than their counterparts.

The odds of developing RR TB among study participants who had a history of TB treatment were 2.05 (95% CI: 1.34, 3.15) times higher than those study participants who had no previous TB treatment. This could be the fact that the history of prior treatment focusing on default letting bacilli strains adapt RIF which provides the chance of bacilli strains mutating (56) and multiplying and finally causing RR TB.

The findings of this research play a crucial role in shaping public health policies, treatment guidelines, and future research directions to effectively address the challenges posed by drug-resistant tuberculosis strains.

It impacts public health policies by informing the development of public health policies aimed at controlling the spread of rifampicin-resistant tuberculosis, particularly drug-resistant tuberculosis at large. For example, public health officials can implement targeted interventions to reduce HIV-TB coinfection and previous TB treatment history and prevent the emergence of drug-resistant strains. Additionally, the findings can help guide the allocation of resources for surveillance, diagnostic testing, and treatment programs for rifampicin-resistant tuberculosis.

The findings of this research can also impact treatment guidelines for rifampicin-resistant tuberculosis. By identifying specific genetic mutations or markers associated with rifampicin resistance, clinicians can use molecular tests to quickly and accurately diagnose drug-resistant strains. This information can help healthcare providers tailor treatment regimens to target the specific resistance mechanisms present in individual patients, leading to more effective and personalized treatment strategies. Finally, the findings of this research may highlight gaps in current knowledge that warrant further investigation, such as understanding the mechanisms underlying the development of drug resistance or exploring potential interventions to mitigate the spread of drug-resistant strains.

## Recommendation

Our recommendation regarding to one out of every twenty-five TB patients experiencing RR TB.

*Enhanced Screening*: Implementing improved screening techniques to detect persons with RR tuberculosis early in their disease progression. This might include routine medication susceptibility testing for all TB patients in order to discover rifampicin resistance early on and commence appropriate treatment.
*Capacity Building*: Strengthening laboratory capacity to perform drug susceptibility testing for rifampicin and other anti-TB drugs. This will ensure timely and accurate diagnosis of drug-resistant TB cases, enabling healthcare providers to deliver targeted treatment interventions.
*Patient Education*: Providing comprehensive education to TB patients about the importance of completing treatment, adherence to medication regimens, and the risks associated with drug resistance. Empowering patients with knowledge can improve treatment outcomes and reduce the spread of resistant strains.
*Contact Tracing*: Implementing robust contact tracing programs to identify individuals who may have been exposed to RR TB cases. This will help prevent further transmission of drug-resistant strains and facilitate early detection and treatment of latent infections.

### Our recommendation depending on the TB-HIV coinfection

*Integrated Screening and Management*: Implementing integrated screening programs to detect people with tuberculosis and HIV infections early on, ensuring quick diagnosis and appropriate treatment.
*Optimized Treatment Regimens*: Individuals with TB-HIV coinfection should have treatment regimens tailored to successfully address both disorders through combination therapy that takes into account potential drug interactions between anti-TB medications and antiretroviral therapies used to treat HIV.
*Regular Monitoring*: Individuals with TB-HIV coinfection should be regularly monitored throughout their treatment to assess response to medication, detect early indicators of treatment failure or drug resistance, and change treatment plans as appropriate.
*Adherence Support*: Providing comprehensive support services to promote treatment adherence among individuals with TB-HIV coinfection, including counseling, education on the importance of completing treatment regimens, and addressing any barriers to adherence that may arise due to the complexity of managing both conditions concurrently.
*Infection Control Measures*: Strengthening laboratory capacity for drug susceptibility testing of rifampicin and other anti-TB drugs. This would enable the timely and accurate diagnosis of drug-resistant tuberculosis cases, allowing healthcare providers to give tailored treatment strategies.
*Research and Surveillance*: Conducting additional research to have a better understanding on the impact of TB-HIV coinfection on the development of rifampicin resistance, as well as to look into novel approaches to improving outcomes for people affected by both conditions, such as evaluating new diagnostic tools or developing targeted interventions to address specific challenges associated with dual infection.

### Our recommendation focusing on having history of previous TB treatment

*Drug Susceptibility Testing*: Conducting drug susceptibility testing for individuals with a history of previous TB treatment to identify any drug-resistant strains early on, which will help guide the selection of appropriate treatment regimens and avoid the use of ineffective drugs.
*Individualized Treatment Plans*: Developing individualized treatment plans for individuals with a history of previous TB treatment and rifampicin resistance. It may be by using alternative anti-TB medications or combination therapy based on the results of drug susceptibility testing to ensure effective treatment.
*Close Monitoring*: Monitoring individuals with a history of previous TB treatment and rifampicin resistance closely throughout their treatment to assess response to therapy, detect any signs of treatment failure or drug resistance early, and adjust treatment plans as needed.
*Adherence Support*: Providing comprehensive support services to promote treatment adherence among individuals with a history of previous TB treatment and rifampicin resistance. This can include counseling, education on the importance of completing treatment regimens, and addressing any barriers to adherence that may arise due to previous treatment experiences.
*Health System Strengthening*: Strengthening health systems to improve TB control programs, enhancing surveillance mechanisms for monitoring drug resistance, and ensuring access to quality diagnostic tools and effective treatments for individuals with a history of previous TB treatment and rifampicin resistance.

## Conclusion

RR TB was one of the emerging challenges among TB patients, which affected approximately one out of every twenty-five TB patients. Having TB-HIV coinfection and history of previous anti-TB treatment were the significant factors associated with RR tuberculosis.

## Data availability statement

The original contributions presented in the study are included in the article/Supplementary material, further inquiries can be directed to the corresponding author.

## Author contributions

MB: Conceptualization, Data curation, Investigation, Methodology, Software, Supervision, Writing – original draft, Writing – review & editing. GB: Conceptualization, Data curation, Investigation, Methodology, Software, Supervision, Writing – original draft, Writing – review & editing. SJ: Conceptualization, Formal analysis, Investigation, Project administration, Resources, Software, Writing – original draft, Writing – review & editing.
