# Supplementary material for: Molecular detection of rifampicin-resistant Mycobacterium tuberculosis by polymerase chain reaction in Ethiopia: a systematic review and meta-analysis
Source: Front Med (Lausanne). 2024 Jun 7;11:1319845. doi: 10.3389/fmed.2024.1319845 (PMC11190194; doi:10.3389/fmed.2024.1319845)
Supplement: Supplementary file 1 [file Table_1.DOCX]

**Study Quality Assurance**

| **Authors name** | **Year of Publication** | **Region** | **Study Area** | **Selection 1) (1 point)**  **Representativeness of the sample** | **Selection 2) ((1 point)**  **Sample size justified and satisfactory** | **Selection 3)(1 point)**  **None response rate** | **Selection 4)(2 point)**  **Validated Measurment tool** | **Comparability**  **Comparability (2 point)** | **Outcome 1) (2 point)**  **Outcome Assessment described** | **Outcome 2) (1 point)**  **Statistical test used described** | **Score** |
| --- | --- | --- | --- | --- | --- | --- | --- | --- | --- | --- | --- |
| Araya Gebreyesus Wasihun etal | 2021 | Amhara | Dessie … | 1 | 1 | 1 | 2 | 2 | 2 | 1 | 10 |
| Sebsib Selfegna *etal* | 2022 | Amhara | Shewa | 1 | 1 | 0 | 2 | 2 | 2 | 1 | 9 |
| Wondemagegn Mulu *et al* | 2017 | Amhara | Debre Markos | 1 | 1 | 1 | 2 | 2 | 2 | 1 | 10 |
| Waganeh Sinshaw *etal* | 2019 | Addis Ababa | Addis Ababa | 1 | 1 | 1 | 2 | 2 | 2 | 1 | 10 |
| Balew Arega *et al* | 2019 | Addis Ababa | Addis Ababa | 1 | 1 | 1 | 2 | 2 | 2 | 1 | 10 |
| Feleke Mekonnen *et al* | 2015 | Amhara | Metema | 1 | 1 | 1 | 2 | 2 | 2 | 1 | 10 |
| Tsehaye Asmelash Dejene *et al* | 2017 | Tigray | Adwa | 1 | 1 | 1 | 2 | 2 | 2 | 1 | 10 |
| Shambel Araya *et al* | 2022 | Addiss Ababa | Merkato | 1 | 1 | 1 | 2 | 2 | 2 | 1 | 10 |
| Daniel Gebretsadik *et al* | 2022 | Amhara | Ataye | 1 | 1 | 1 | 2 | 2 | 2 | 1 | 10 |
| Gebremedhn Bizayen Gebrehiwet etal | 2018 | Afar | Dubti | 1 | 1 | 1 | 2 | 2 | 2 | 1 | 10 |
| Wakuman Taye etal | 2021 | Benishangul-Gumuz | Bale zone | 1 | 1 | 1 | 2 | 2 | 2 | 1 | 10 |
| Tibebu Kassa | 2003 | Amhara | Debark | 1 | 1 | 0 | 2 | 2 | 2 | 1 | 9 |
| Awoke Derbie etal | 2016 | Amhara | Debre Tabor | 1 | 1 | 1 | 2 | 2 | 2 | 1 | 10 |
| Olifan Zewdie etal | 2020 | Oromia | Nekemte | 1 | 1 | 0 | 2 | 2 | 2 | 1 | 9 |
| Kefyalew N Jaleta etal | 2017 | Amhara | Gondar | 1 | 1 | 1 | 2 | 2 | 2 | 1 | 10 |
| Tesfaye Andualem Demissie | 2021 | Amhara | Motta | 1 | 1 | 0 | 2 | 2 | 2 | 1 | 9 |
| Kuma Diriba etal | 2021 | SNNP | Gedeo | 1 | 1 | 0 | 2 | 2 | 2 | 1 | 9 |
